# New hyaluronan-based biomatrix for 3-D follicle culture yields functionally competent oocytes

**DOI:** 10.1186/s12958-022-01019-9

**Published:** 2022-10-10

**Authors:** Nina Desai, Maribeth Spangler, Vaani Nanavaty, Arsela Gishto, Alyssa Brown

**Affiliations:** Department of OB/GYN and Women’s Health Institute, Cleveland Clinic Fertility Center, Cleveland Clinic Foundation, Beachwood, OH USA

**Keywords:** Follicle, Ovary, Oocyte, *In vitro* maturation, Biomaterials, 3D printing, Hydrogel, Ovarian tissue engineering, Cell encapsulation, Fertility preservation

## Abstract

**Background:**

Encapsulation of follicles within a biomatrix is one approach to maintaining 3-D follicle architecture during culture. Hyaluronan is one component of the natural extracellular matrix (ECM) that provides support to cells in vivo. This report describes the application of a novel tyramine-linked hyaluronan for 3-D *in vitro* follicle culture and the production of developmentally competent metaphase II oocytes.

**Materials and Methods:**

Enzymatically isolated mouse preantral follicles or follicle clusters (FL-C) from fresh or vitrified ovaries were encapsulated in 3 mg/ml of hyaluronan gel (HA). Follicle growth, antrum formation and meiotic maturation to metaphase II oocytes was monitored. Chromatin staining was used to assess GV oocyte progression towards meiotic competence. Functional competence of *in vitro* matured (IVM) oocytes was evaluated by *in vitro* fertilization and ability to develop to blastocyst. Modifying the HA gel by inclusion of laminin (HA-LM), mouse sarcoma extracellular matrix (Matrigel;HA-MG) or placental extracellular matrix (HA-PM) was also tested to see if this might further enhance IVM outcomes.

**Results:**

A total of 402 preantral follicles were cultured in HA gel. After hCG trigger, 314 oocyte-cumulus complexes ovulated from the embedded follicles. Meiotic maturation rate to the metaphase II stage was 73% (228/314). After insemination 83% (188/228) of IVM oocytes fertilized with a subsequent blastulation rate of 46% (87/188). A pilot transfer study with 3 recipient mice resulted in the birth of a single pup. HA gel supported individually isolated follicles as well ovarian tissue fragments containing clusters of 6–8 preantral follicles. Meiotic maturation was lower with FL-clusters from vitrified versus fresh ovaries (34% and 55%, respectively; p < 0.007). Modification of the HA gel with ECMs or laminin affected antrum formation and follicle retention. Maturation rates to the metaphase II stage were however not significantly different: 74% for HA gel alone as compared to HA-LM (67%), HA-MG (56%) and HA-PM (58%).

**Conclusion:**

Hyaluronan gel is an effective and versatile extracellular matrix based biomaterial for 3-D culture of ovarian follicles. This culture model allowed ovulation of functionally competent metaphase II oocytes, capable of fertilization, genomic activation and blastulation. Future testing with human follicles that require longer *in vitro* culture times should be considered.

## Introduction

Tremendous progress has been made over the past three decades in treating infertility. Advances in ovarian stimulation regimens, embryo culture methodology, and cryopreservation techniques have all resulted in a rise in implantation rates. For cancer patients, these improvements have increased the odds of a future pregnancy if they are able to successfully complete fertility treatment before chemo or radiation therapy is initiated. Often however, ovarian tissue cryopreservation (OTC) is still the only option for preserving fertility in adult women where immediate initiation of cancer therapy is imperative or in young pre-pubertal girls. The obvious next question is how best to use this frozen tissue once the patient is in remission. Ovarian tissue transplantation was considered research until 2019, when the American Society of Reproductive Medicine deemed it an acceptable clinical tool for establishing pregnancies. To date, worldwide there have been over 130 children born from ovarian tissue transplantation [1, 2]. OTC also allows the patient to retain endocrine function. A non-surgical approach to deriving mature oocytes *in vitro* from cryopreserved ovarian tissue is however still highly desirable, especially with certain types of cancer. The potential risk of re-introducing malignant cells with ovarian transplantation could be eliminated by follicle culture and *in vitro* maturation of oocytes for subsequent IVF.

Much research has therefore focused on understanding the complex regulatory mechanisms for follicle recruitment and growth. The follicular unit consists of somatic cells and the oocyte itself. Primordial follicles from the ovarian cortex are recruited into a growing cohort of “primary” follicles. With continued growth and increasing exposure to FSH, follicles progress to the preantral and antral stages. Bidirectional communication between surrounding granulosa cells and the oocyte is required for appropriate nuclear and cytoplasmic maturation [3–8]. Conventional 2-D culture systems are inadequate for extended time *in vitro* as they allow follicle attachment, flattening and ultimately the migration of granulosa cells away from the oocyte. Compelling evidence with a variety of cell types suggests that cell growth, signaling and response to stimuli in 3-D culture models provide a more physiologic environment [9–14]. Taking into consideration the complexity and critical nature of interactions between oocyte, granulosa and thecal cells, culture methodologies tailored to maintain 3-D follicle architecture have garnered a lot of interest.

The first live offspring from *in vitro* matured mouse follicles was achieved using collagen impregnated gels in 1989 by Epping et al. [15]. Since then numerous culture methods and matrices have been explored for 3-D follicle culture (reviewed [16–22]. Extensive work has been published using alginate for 3-D culture of mouse and human follicles [23–26]. Other biomaterials have not received as much attention and need further exploration.

Hyaluronan is a naturally occurring glycosaminoglycan and also the major component of the in vivo extracellular matrix (ECM) that gives structural support to cells, as well as biochemical signals for tissue morphogenesis and differentiation. For this reason, it is an attractive option as a biomaterial for 3-D culture of follicles. Earlier work from our laboratory tested a novel tyramine-linked hyaluronan (HA) hydrogel for its ability to support growth of mouse preantral follicles. We experimented with different concentrations of gel and various embedding methodologies. Cultivated follicles showed progressive *in vitro* growth with rising estradiol levels over a period of 10-12 days and maintained their spheroid architecture. This HA gel supported antrum formation, ovulation with hCG trigger and maturation of enclosed GV oocytes into mature metaphase II oocytes [27]. The current report expands on our initial studies with this hyaluronan matrix for 3-D follicle culture and *in vitro* oocyte maturation (IVM). The primary objective of this study was to determine if this biomaterial supports the production of developmentally competent metaphase II oocytes capable of fertilization and growth to blastocyst. We also document the first transfer attempt and pregnancy using these IVM-derived blastocysts. Our secondary objective was to test other ECM components in conjunction with this HA gel to further improve on this 3-D culture system.

## Methods

### Animals

B6D2F1 mice (cross between female C57BL/6 and male DBA/2 males) were purchased from Charles River Laboratories, Wilmington, MA and maintained as a breeding colony. Animals were housed at the Cleveland Clinic’s Animal Care facility within an environment of controlled temperature and lighting (12 h dark: 12 h. light). Animals were handled according to NIH guidelines and following IACUC protocols of the Cleveland Clinic. Ovaries were dissected from 12-14-day old female pups for follicle isolation. Mice were sacrificed by cervical dislocation. Each experimental run consisted of ovaries from 3-6 pups. Male mice at age 6-8 weeks were used for *in vitro* fertilization runs. Sperm were isolated from the epididymis and used for oocyte insemination.

### Follicle isolation and culture

Ovaries were placed in an organ culture dish containing pre-warmed Leibovitz-15 medium (Invitrogen; Carlsbad, CA) with 0.1% collagenase (Type I; Worthington Biochemical, Lakewood, NJ). The dish was incubated on a laminar flow bench top at 37 °C for 30 min. After 30 min, ovaries were rinsed free of enzyme and placed in a 60 mm dish containing L15 medium with 0. 1% FBS. Follicles were released from the ovaries by gentle pipetting using a 200-ul Eppendorf pipettor. Using a dissecting microscope, released follicles were collected using a glass micropipette. Follicle clusters (FL-C) were isolated by teasing apart the collagenase treated ovaries with needles. Follicle clusters containing 6-8 intact preantral follicles were selected for culture. Follicle clusters were washed three times in culture medium to remove any traces of L-15 or collagenase.

Culture was performed in α-modified Minimum Essential Medium (α-MEM; Invitrogen) supplemented with 5% fetal bovine serum, 100 mIU/ml FSH,10 mIU/ml LH, ITS (10 µg/ml insulin, 5 µg/ml transferrin and 5 ng/ml selenium; Invitrogen). Preantral follicles ( 100-140 µm, oocyte < 65 µm) with a centrally located oocyte, surrounded by 2 or more layers of granulosa cells and enclosed within an intact basement membrane were selected for experiments. Follicle culture was performed in the 8-well GPS dish (LifeGlobal) with 100 ul of follicle culture medium in each well and an oil overlay.

### Follicle encapsulation

A novel tyramine-linked hyaluronan (HA) hydrogel developed at the Cleveland Clinic Department of Biomedical Engineering (Corgel ® BioHydrogel;Lifecore,MN) was used for follicle encapsulation (Desai et al. 2012). Tyramine-substituted sodium hyaluronate powder (250 mg) was rehydrated with 25 mls of horseradish peroxidase (HRP) in 10 mls of PBS. Aliquots of this stock activated HA (10 mg/m) were stored at -4 °C. For follicle experiments. HA was prepared by diluting to a concentration of 3 mg/ml in Global medium (Life Global; Guilford, CT). For embedding, one microliter of 0.03% hydrogen peroxide was mixed into 25 µl of activated HA gel to initiate cross linking and gel formation. Working very quickly, two drops of HA gel (~ 8-10 µl each) were placed in individual wells of a specialty 60 mm IVF culture dish with 8 × 100 µl wells arranged in a circular pattern (GPS dish; LifeGlobal). Using a glass micropipette, 4 preantral follicles were immediately deposited into the center of each gel bead. Minimizing the tracking of culture medium to prevent gel dilution was critical to keeping the follicles properly embedded. Embedding steps were carried out at 37 °C in a laminar flow hood. After three minutes, 100 µl of pre-equilibrated culture medium was added to each well. Once all 8 wells were loaded, the dish was overlaid with warmed oil and follicles were cultured at 37 °C with 6% CO in air for 12 days.

Inclusion of extracellular matrix proteins with hyaluronan during encapsulation was also explored to determine if this provided additional benefit. Laminin from human placenta (LM, Sigma-Aldrich; St. Louis, MO) was added at concentrations of 50, 100 and 200 µg/ml to HA. HA was also tested with addition of 100 µg/ml of Matrigel (MG, Corning Life Sciences; Tewksbury,MA) purified from Engelbreth Holm Sarcoma or 100 µg/ml of human placental extracellular matrix (PM, Corning Life Sciences).

### Follicle culture, assessment and oocyte maturation

During the *in vitro* culture interval (IVC) medium was exchanged every other day by replacing one half of the culture volume with fresh equilibrated medium. Follicles were visualized using an Olympus IX70 inverted microscope equipped with Hoffman modulation contrast optics system, a high definition camera and image capture software. Follicle morphology was assessed at medium exchanges. Culture wells were monitored for continued follicular growth and antrum formation. Follicles appearing dark/degenerative or where the oocyte was no longer enclosed within the granulosa cell layer were classified as non-viable. Follicle and oocyte diameters were determined by taking the average of two perpendicular measurements of diameter. Follicle diameters were measured from the outer edge of the thecal cell layer. Oocyte diameter was measured at the outset of the experiment and after the final maturation step. Additional oocyte measurements were taken during the IVC if the oocyte could be clearly visualized. Antrum formation was identified by appearance of a visible cavity or clearing amidst the granulosa cells surrounding the oocyte.

Antrum formation marked the end of the follicular growth phase. Final *in vitro* maturation was triggered by exposure of the follicles to culture medium supplemented with 1.5 IU/ml of human chorionic gonadotrophin (HCG), and 5 ng/ml of epidermal growth factor (EGF) (R&D Systems, Minneapolis, MN). Maturation was allowed to proceed overnight for 16-18 h. Ovulated oocyte complexes (OC) were easily visualized the next morning above the HA gel. The OC were collected and briefly treated with hyaluronidase (10 U/ml) to assist in denuding the oocytes free of cumulus cells to allow assessment of nuclear status. After rinsing to remove residual enzyme, the oocytes were placed in 5 µl drops of fresh medium, separated according to initial well number. Oocytes were assessed at 400X magnification. Each oocyte was photographed and its meiotic status documented. Live imaging of metaphase II oocytes was performed to identify a meiotic spindle and assess chromatin organization using the Oosight Imaging system (CRI; Hopkinton, MA) and polarized light. Retardance levels were recorded to quantify spindle birefringence.

## Oocyte chromatin staining

Chromatin staining in GV oocytes was used to monitor progression to meiotic competence. Live staining was performed on GV oocytes with Hoechst 33342 (Sigma-Aldrich; 50 ng/ml) at start of culture on day 0 (D0) and after antrum formation prior to hCG trigger (D 9-10). During oocyte growth, chromatin in the GV oocyte condenses to form perinucleolar rings. GV oocytes were classified according to whether the nucleolus was surrounded by a ring of chromatin (SN) or non-surrounded (NSN) [28].

Morphology of meiotic spindles in metaphase II oocytes recovered from ovulated complexes was examined using immunofluorescent staining. Oocytes were fixed in 2% paraformaldehyde in phosphate buffered saline with 0.1% Triton-X for 30 min at 37 °C. Oocytes were then blocked with 10% mouse serum/1% human serum albumin for an hour at 37 °C before staining overnight with a 1:100 dilution of fluorescein (FITC) labelled anti- alpha and anti- beta tubulin antibody (Sigma-Aldrich) The next morning, oocytes were rinsed and stained with propidium iodide (1 ug/ml) for 45 min at 37 °C. Meiotic spindles in oocytes were visualized using the Keyence BTX microscope and fluorescent microscopy.

### *In vitro* Fertilization (IVF) and embryo transfer

*In vitro* maturation of oocytes in HA embedded follicles was initiated as described above. For IVF experiments, oocytes were not denuded. OCs were collected from embedded follicles 15 h after exposure to maturation medium containing hCG. The oocyte complexes were placed in 500 µl of TYH medium (CytoSpring; San Francisco, CA) supplemented with 1 mM glutathione- reduced (GSH, Sigma-Aldrich) in the center well of an organ culture dish with an oil overlay and incubated at 37 °C. Epididymal sperm isolated from male mice were allowed to capacitate in TYH-GSH medium for one hour at 37 °C with 6% CO_2_. The oocyte dish was inseminated with 4 million sperm. After 4-5 h, oocyte-cumulus complexes were transferred to KSOM-AA medium (CytoSpring) supplemented with 1 mM GSH and 25 ng/ml of Stem cell factor (SCF, PeproTech; Rocky Hill,NJ) and cultured overnight. The next morning, cleaving 2-cell embryos as well as any oocytes displaying 2 pronuclei were counted as fertilized and moved to fresh pre-equilibrated KSOM-AA medium with SCF.

Blastocyst formation was assessed three days later. Total cell number in blastocysts was determined by Hoechst staining of chromatin in cell nuclei. Blastocysts were also cryopreserved by vitrification for future transfer experiments. Methodology for blastocyst vitrification using the Rapid i carrier and warming has been previously described [29].

Transfer experiments could not be conducted at our clinical laboratory site since we did not have the facilities to house post-surgical animals. Vitrified blastocysts were therefore sent to Charles River Laboratories (CRL) to attempt transfer of IVM derived blastocysts. Blastocysts were warmed and embryo recovery and survival rates were calculated. Embryos were held in the incubator for 15 min before transfer. Embryo transfers were conducted via surgical uterine transfer into 1.5 dpc recipient CD-1 mice produced by natural mating on natural estrus cycles following all CRL approved surgical operating protocols. One recipient received 14 embryos via bilateral uterine transfer. The remaining two recipient mice had 16 embryos transferred via bilateral uterine transfer.

### Ovarian tissue vitrification/warming

Excised ovaries were vitrified using ethylene glycol/dimethyl sulfoxide (EG/DMSO) as the cryoprotectant. [30]. Vitrification solutions (VS) were formulated in L-15 medium containing 20% Synthetic Serum Substitute (Irvine Scientific; Irvine, CA). Ovaries were equilibrated step-wise in chilled VS solutions with increasing concentrations of the cryoprotectant agent: 5% for 5 min, 10% for 5 min, 15% for 10 min and finally 20% EG/DMSO. All steps were performed at 4 °C. Ovaries (2-3) were then loaded on a nylon mesh and plunged into a specially vented cryovial filled with liquid nitrogen. Ovaries were warmed by equilibrating step-wise in medium containing 0.5, 0.25 and 0.125 M sucrose for 5 min each. Individual follicles and follicle clusters were isolated by collagenase digestion as described earlier or by mechanical dissection using needles.

### Hormone secretion

Estradiol concentrations in conditioned media collected from follicle cultures at various time points during the *in vitro* culture interval were assessed. At each time point, media samples from four wells each containing 4 follicles were pooled and estradiol concentrations per well were determined using an enzyme-linked immunoassay (ELISA) kit (Calbiotech; El Cajon, CA). All measurements were done in triplicate. Hormone secretion per follicle was calculated. The limit of sensitivity for the estradiol assay was 3 pg/ml.

### Outcome parameters and data analysis

Follicle viability was determined by monitoring morphology, changes in follicle/oocyte diameters, antrum formation and ovulatory response after administration of hCG trigger. Functional competence of *in vitro* matured oocytes was assessed based on presence of GV oocytes with SN chromatin staining pattern and the resumption of meiosis in ovulated oocytes. Percentage of recovered oocytes undergoing germinal vesicle breakdown (GVBD) and forming metaphase II oocytes were calculated in each experiment. Competence of HA-derived IVM oocytes to undergo fertilization, and develop to blastocysts was analyzed.

All statistical calculations were done with the JMP 14 software package (SAS Institute Cary, NC). Categorical data were analyzed using the chi square test for independent samples. Statistical differences in follicle/oocyte size and estradiol secretion were assessed by variance analysis with one-way ANOVA followed by the Tukey test for multiple comparisons. Differences were considered to be significant if *p* values were < 0.05.

## Results

### Follicle morphology during 3-D culture in HA gel

**Fig. 1:**
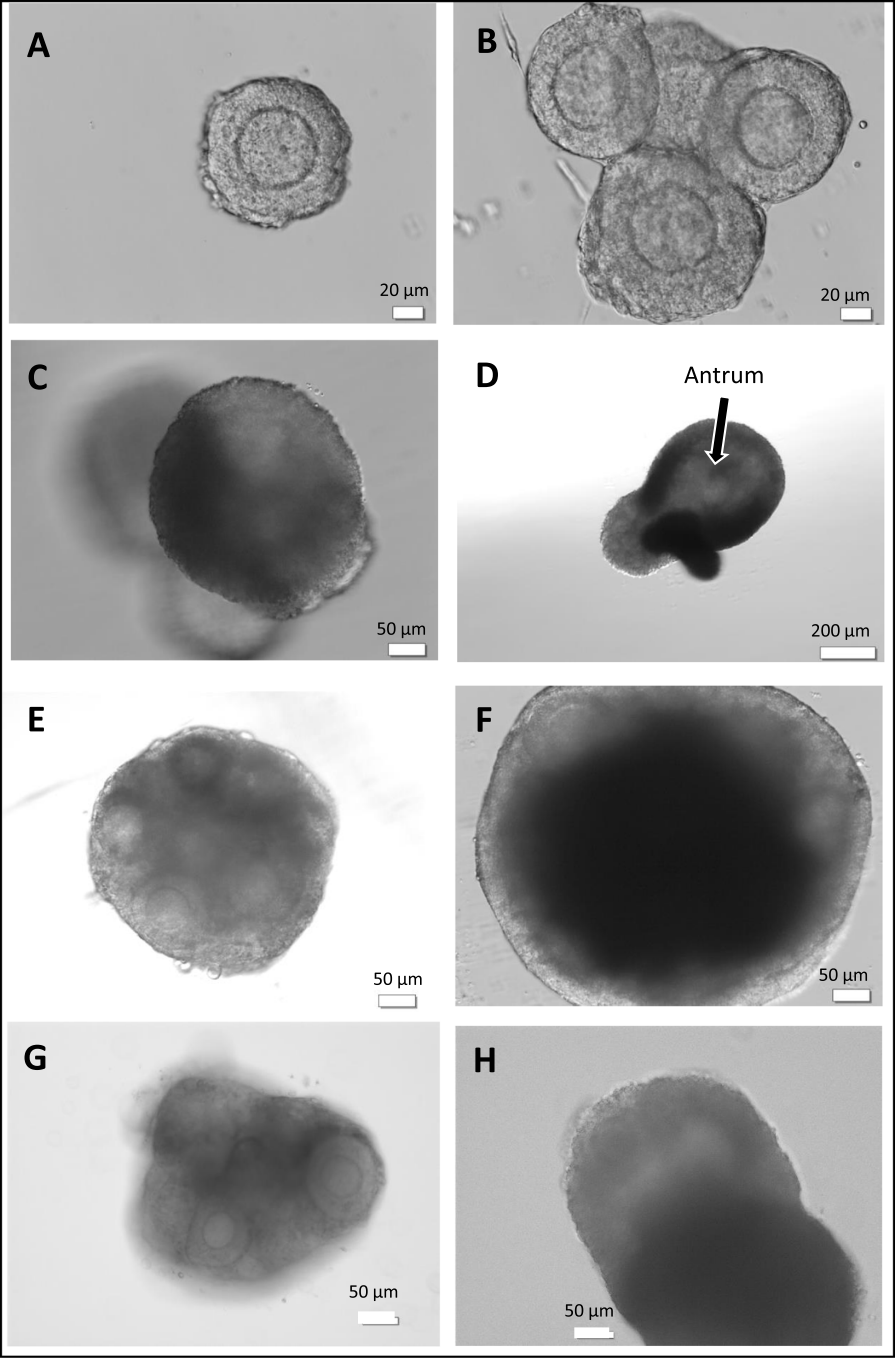
**A** Preantral follicle with 2–3 layers of granulosa cells collected after collagenase digestion of ovary (**B**) Individually isolated follicles seeded in HA gel on day 2 of culture. Follicles tended to aggregate during gelling process. **C** Day 4 of culture. Follicles growing in different planes within HA gel (**D**) HA-encapsulated on day 9 of culture. Antrum formation discernible (**E**, **F**) FL-cluster from fresh ovary embedded in HA gel shown at start of culture and on day 6. Granulosa cell proliferation evident and FL-cluster takes on an”organoid” appearance (**G**, **H**) FL-cluster mechanically isolated from vitrified ovary shown on day 2 and day 8 of culture

Intact secondary follicles with centrally located oocytes surrounded by 2-3 layers of granulosa cells were selected for encapsulation in HA gel. Pre-antral follicles measured 140.4 ± 29.1 µM with oocyte diameters of 63.5 ± 4.0 µM at time of seeding. Figure 1 A-D depict HA gel drops seeded with individually isolated follicles from the ovarian digest. Follicle morphology and morphological changes during *in vitro* growth are shown. The HA-embedded preantral follicles maintained their 3-D spheroid architecture throughout the *in vitro* culture interval. Between Day 1 and 4, there was a 1.4 fold increase in follicle size with a mean diameter measuring 201.6 ± 28.5 µm. The basal lamina with thecal cell layer was evident in most follicles. Antral cavity formation by encapsulated follicles was generally initiated by day 7 or 8 of culture. Ovulation was typically triggered between days 9-12, when ~ 50% of cultured follicles displayed an antrum, as shown in Fig. 1D. The average oocyte diameter in collected metaphase II oocytes was 90.2 ± 8.0 µM.

HA gel also supported the culture of ovarian tissue fragments. Figure 1E, F illustrate growth of follicle clusters (FL-cluster) isolated from fresh ovaries, containing 6-8 intact preantral follicles. The last two panels (Fig. 1G, H) show images of FL-clusters from vitrified ovaries. Follicular architecture was preserved within these FL-clusters. Granulosa cells around the oocytes proliferated extensively. Within four days of culture it was difficult to discern individual follicles within the cultured fragments Antrum formation was however still easily visualized. Mature oocytes could be retrieved from these “organoid” like follicle constructs after hCG trigger. No differences were observed in oocyte morphology or final diameter between oocytes derived from individually isolated follicles or FL-clusters. Enzymatic treatment of ovaries followed by mechanical manipulation to obtain either individual follicles or FL- clusters was the most efficient means for handling fresh ovaries. In contrast, with vitrified ovaries, attempts to isolate intact individual follicles using collagenase were not satisfactory. All too often the oocytes were released or damaged. We found the best approach was to handle the tissue minimally. Mechanical dissection of the vitrified/warmed ovaries to obtain FL-clusters that could subsequently be encapsulated in HA-gel appeared to be the optimal method to derive mature oocytes. Embedding the FL-clusters also helped to keep the follicles intact and prevented the premature ovulation of oocytes before the end of the *in vitro* culture interval.

Table 1 compares *in vitro* follicle growth and maturation data for oocytes from fresh and vitrified ovaries. With fresh ovaries, the oocyte ovulation and the final rate of oocyte maturation were similar regardless of whether follicles were cultured individually or as a cluster. In contrast, meiotic maturation to the metaphase II stage was significantly lower with the FL-clusters isolated from vitrified ovarian tissue (34%) as compared to fresh FL-cluster and FL-Individual (55%and 59%, respectively; *p* = 0.007).

**Table 1:** Comparison of *in vitro* growth of HA encapsulated follicles from fresh versus vitrified ovaries

	**Fresh**	**Fresh**	**Frozen**	***P*-Value**
	**FL-Isolated**	**FL-Cluster**	**FL-Cluster**	
	130	154	69	
Antrum formation	51%(66/130)	53% (81/154)	-	
Ovulated complexes after hCG	71% (92/130)	66% (101/154)	93% (64/69)	0.0001
GVBD (MI formation)	30% (28/92)	28% (28/101)	52% (33/64)	0.004
Maturation to MII	59% (54/92)	55% (56/101)	34%( 22/64)	0.007

This table contrasts outcomes between fresh and frozen ovaries. These data show that all outcome parameters for FL-Isolated and FL-Clusters from fresh ovaries were similar but differed significantly with results from frozen ovaries. The final maturation rate to MII was significantly lower with FL-Clusters from frozen ovaries versus fresh FL-Isolated or fresh FL-cluster

### Morphologic assessment of oocyte competence

**Fig. 2:**
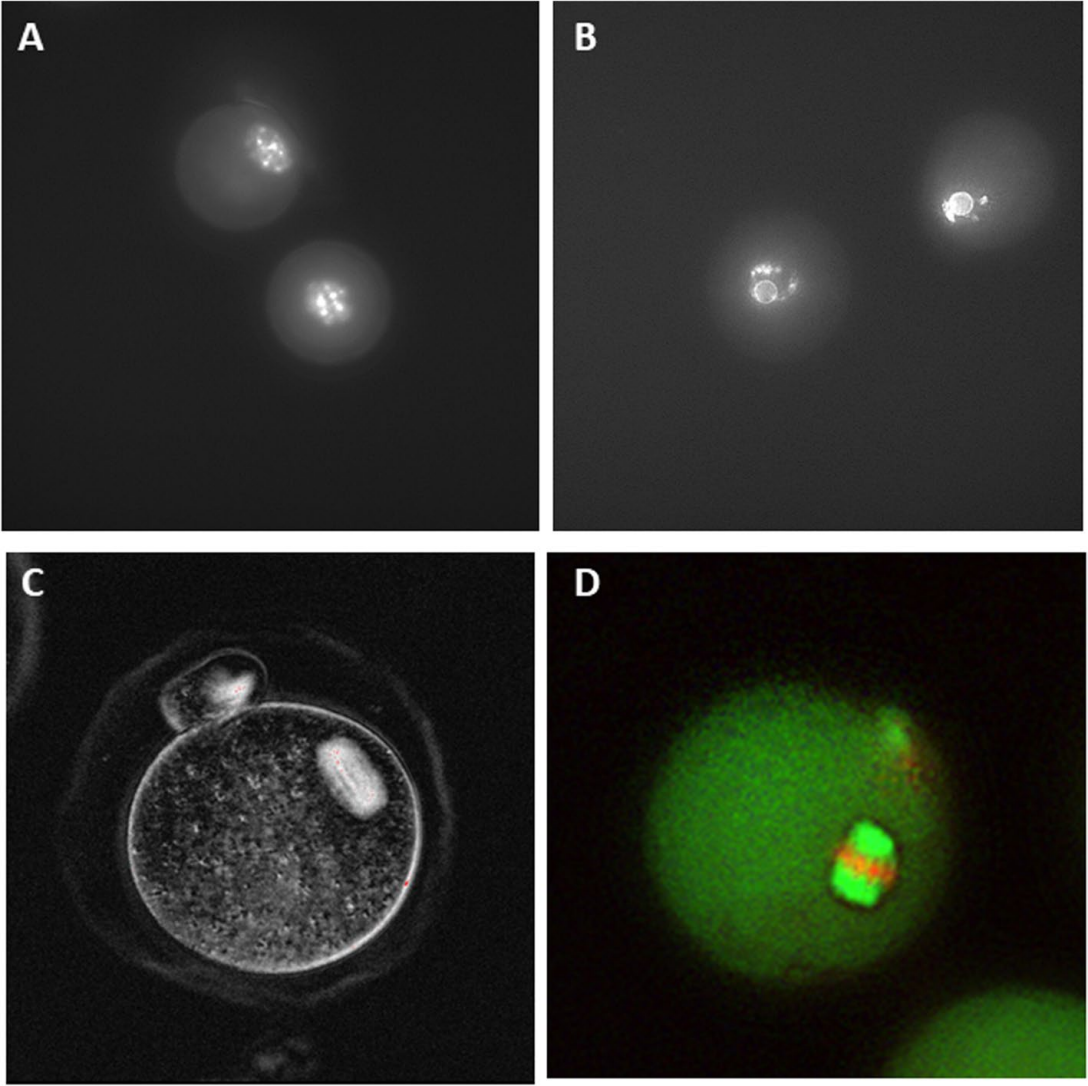
**A** Chromatin staining at outset of culture showing GV oocyte with non-surrounded chromatin staining (NSN) pattern (**B**) oocyte from growing follicle at time of antrum formation on day 9. GV oocyte with SN staining pattern. Chromatin condensed and forming ring around the nucleolus (**C**) Live imaging of metaphase II oocyte and spindle using polarized light and birefringence (**D**) Immunofluorescent staining of metaphase II oocyte showing normal meiotic spindle with fluorescent microtubules running to poles and chromosomes aligned on equatorial plate. 400 × magnification

Changes in GV oocyte chromatin organization from the preantral to the antral follicle stage were evaluated. At outset of follicle culture, all of the isolated GV oocytes exhibited the NSN staining pattern, with the nucleolus not surrounded by chromatin. Chromatin pattern was re-assessed when antral cavities were observed in 40-50% of follicles. GV oocytes were extracted by pipetting the HA encapsulated follicles. We then classified the oocytes into two groups SN and NSN based on chromatin pattern after Hoechst staining (Fig. 2A, B). Oocyte nuclear chromatin in the GV oocytes had reorganized during the *in vitro* culture interval such that 83% of oocytes (38/46) recovered from follicles now exhibited the SN configuration. Distinct perinuclear rings were observed surrounding the nucleolus.

We also examined meiotic spindle morphology in the *in vitro* matured metaphase II oocytes. Birefringent spindles were easily visualized upon live imaging of ovulated oocytes with polarized light (Fig. 2C). Immunofluorescent staining was performed on fixed metaphase II oocytes to further assess spindles. Figure 2D shows an example of an oocytes with a normal spindle configuration. Normal meiotic spindles were detected in 72% (28/39) of IVM eggs.

#### Functional competence of oocytes to fertilize and develop to blastocysts

**Fig. 3:**
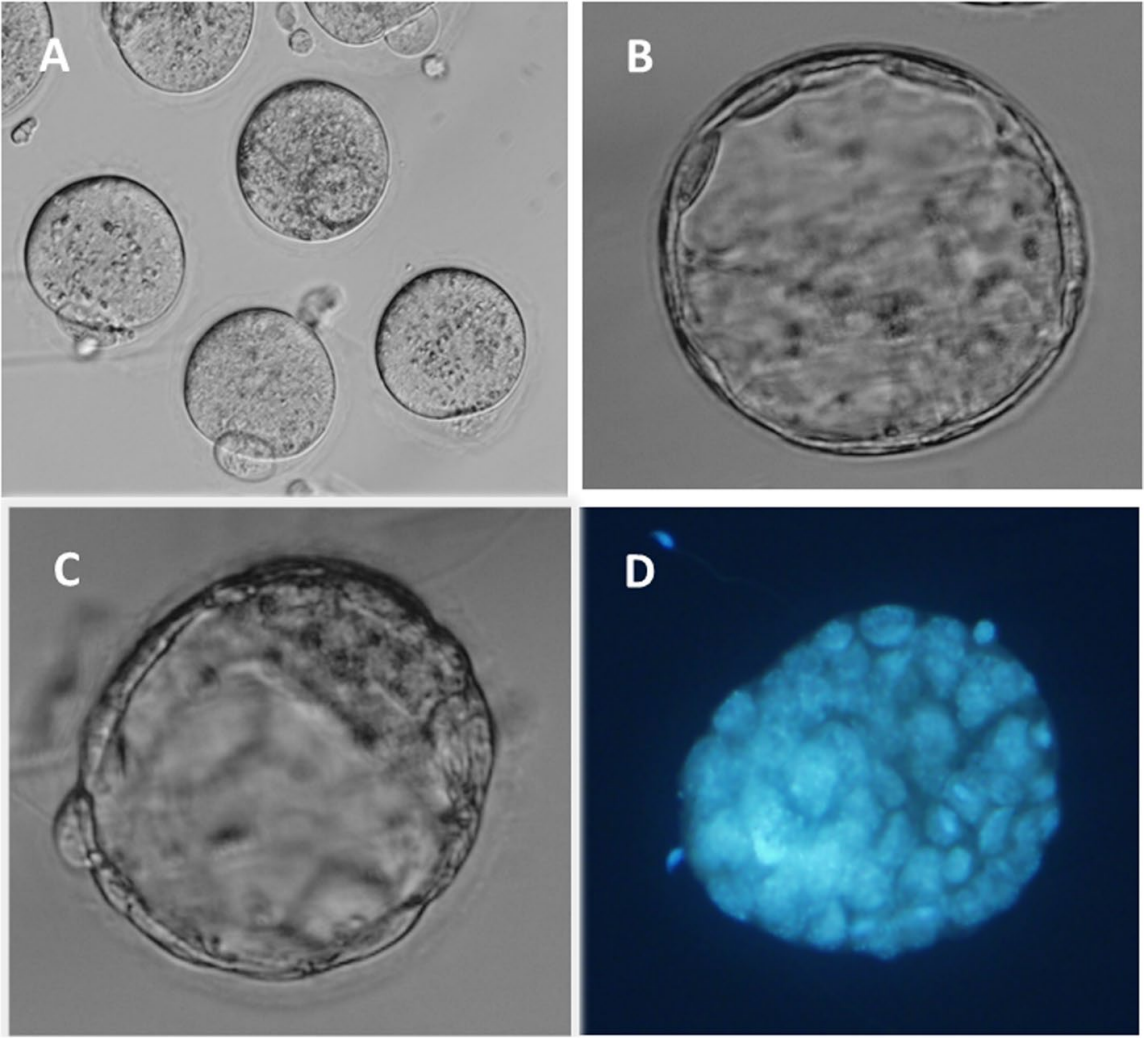
**A** Ovulated oocytes from *in vitro* cultured HA encapsulated preantral follicles prior to IVF insemination (**B**, **C**, **D**) Blastocysts derived from *in vitro* matured oocytes derived from HA gel (**D**) Blastocyst labelled with blue fluorescent nuclear DNA stain to determine blastomere count. 400 × magnification

IVM data from three experiments were pooled. (Table 2). A total of 402 pre-antral follicles were embedded in HA gel and cultured for 9 days until antral follicle formation was observed. Oocyte-cumulus complexes (*n* = 314) were collected after hCG trigger. We tested the competence of *in vitro* matured oocytes to be fertilized and develop to the blastocyst stage. Table 2 summarizes these data. The rate of oocyte maturation to the metaphase II stage was 73%. Our data established that these IVM oocytes (Fig. 3A) were functionally capable of being both fertilized at a high rate (83%) and undergoing embryonic genomic activation, with a blastulation rate of 46%.

**Table 2:** Fertilization and blastocyst formation with *in vitro* matured oocytes derived from HA encapsulated follicles

	**HA-Embedded Follicles**
Follicles cultured	409
Ovulated complexes	314 (77%)
Mature oocytes	228 (73%)
Fertilized oocytes	188 (82%)
Blastocysts	87 (46%)

Data pooled from 3 separate experiments with HA embedded follicles cultured *in vitro* for 10–12 days before final maturation. Mature oocytes were inseminated with sperm. High quality blastocysts were vitrified for a future transfer attempt

A total of 61 expanded blastocysts were cryopreserved for future transfer experiments to ascertain their ability to implant and produce live offspring (Fig. 3B,C,D). Hoechst staining was performed on remaining blastocysts to approximate blastomere number (Fig. 3D). The mean cell count in the assessed blastocysts was 55 ± 7.5. However since the more advanced blastocysts were cryopreserved, this somewhat low overall cell count was not unexpected.

Our pilot study with transfer of vitrified –warmed blastocysts was conducted at Charles Rivers Laboratories. Embryo recovery and survival rates after warming were 82% (50/61) and 92% (46/50), respectively. A total of 46 thawed blastocysts were transferred into three recipients. One of the three recipient mice was observed to be pregnant 18 days post-transfer. A single pup was delivered. The health of the pup could not however be determined as it was cannibalized shortly after birth.

#### Follicle encapsulation and effect of extracellular matrix proteins

In this series of experiments, we investigated modifying the HA gel with extracellular matrix components to determine if this might further enhance IVM outcomes. To this end, we tested laminin as well as two extracellular matrix complexes, one from mouse tumor cells (Matrigel), the other from human placenta. The basic composition of both ECM matrices consisted of collagen IV, laminin, glycosaminoglycans, chondroitin sulfate and growth factors reflective of their tissue of origin.

**Fig. 4:**
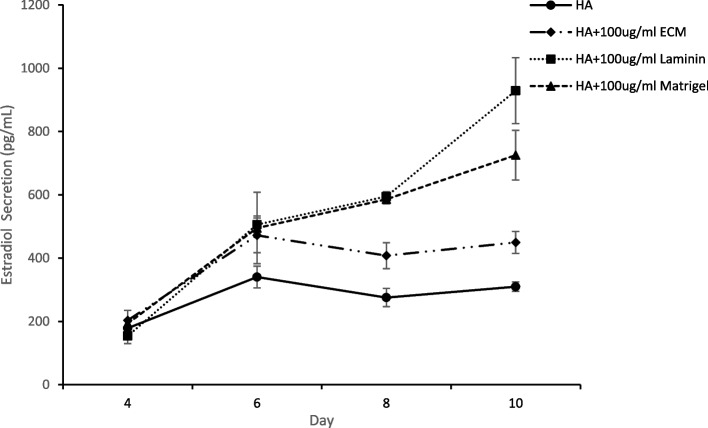
Estradiol secretion per follicle during 3-D culture. Comparison between follicles encapsulated in HA alone versus HA modified with laminin, placental ECM or mouse sarcoma ECM (Matrigel). Estradiol secretion significantly lower in HA alone versus HA with laminin or either ECM (*p* < 0.001) Data is expressed as mean ± SD

Follicles encapsulated in HA-LM, HA-MG and HA-PM exhibited more rapid proliferation of granulosa cells and within a few days of culture it became difficult to visualize the oocytes. Table 3 compares follicular growth and oocyte maturation in HA alone versus in HA modified with LM or ECMs. Significant differences were noted between the treatments in terms of follicle retention in the gel, percent antrum formation and ultimately ovulated complexes after hCG trigger. Preantral follicles encapsulated within HA gel with either type of ECM matrix were better retained in 3-D culture to the end of the growth interval (HA-MG 88%, HA-PM 94% vs HA alone 69%; *p* < 0.005). Significantly lower antrum formation was noted amongst follicles in HA-LM (29%), HA-MG (18%) and HA-PM (26%) as compared to HA alone, 48%; *p* = 0.006). The HA-Matrigel encapsulated follicle treatment group had a lower rate of ovulation (37%) as compared to all three other treatment groups ( 65-67%; *P* = 0.001). The rate of maturation to metaphase II oocytes was 74% for HA gel alone as compared to HA-LM ( 67%), HA-MG (56%) and HA-PM (58%) but differences were not statistically significant. Inclusion of matrix components in the HA gel did however significantly increase overall estradiol secretion measured on day 8 and 10 of culture (Fig. 4). This was likely due to the positive effect of ECM and laminin on granulosa cell proliferation. The highest level of estradiol per encapsulated follicle was with HA-LM (929 ± 104 pg/ml) as compared to HA alone (309 ± 15 pg/ml; *p* < 0.001). But it is important to note that there was no correlation between estradiol secretion and oocyte maturation rates.

**Table 3:** Follicle culture and oocyte maturation in hyaluronan gel modified with laminin or ECMs

	**HA**	**HA-LM**	**HA-MG**	**HA-PM**	***P*-value**
Follicles initially plated	70	76	76	62	
Follicles retained in gel	48 (69%)	58(76%)	67 (88%)^a^	58 (94%)^a^	0.0006
Antrum formation	23 (48%)	17 (29%)^**b**^	12 (18%)^c^	15 (26%)^b^	0.006
Ovulated complexes	31 (65%)	39 (67%)	25 (37%)^a^	38 (66%)	0.001
GVBD to MI (%)	6 (17%)	12 (31%)	11 (44%)	16 (42%)	NS
Maturation to MII (%)	23 (74%)	26 (67%)	14 (56%)	22 (58%)	NS
MII oocyte diameter (µM)	91 ± 3.3	96.5 ± 4.1	89.3 ± 2.8	89.1 ± 2.8	NS

Comparison between follicles encapsulated in hyaluronan (HA) alone versus HA modified with laminin (HA-LM), mouse sarcoma ECM (Matrigel) or placental ECM (HA-PM). Percent MI and MII formation from ovulated eggs was calculated. Significantly different when compared to HA alone

^a^*p* < 0.005

^b^*p* < 0.05

^c^*p* = 0.0006

**Fig. 5:**
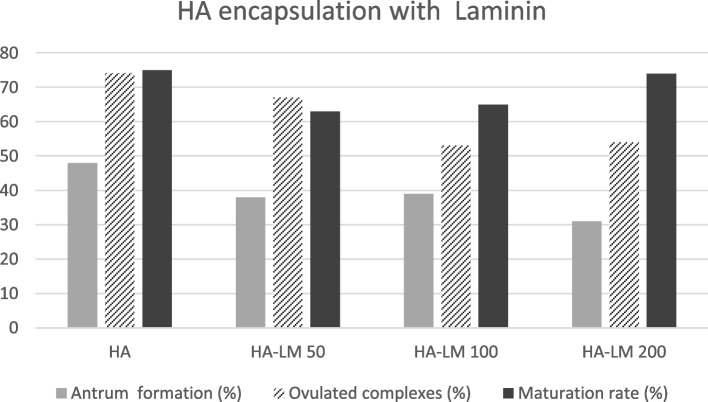
Encapsulation of follicles in HA with varying concentrations of laminin was tested. A total of 245 follicles were embedded as follows: 1-HA alone 2-HA with 50 ug/ml LM, 3- HA with 100 ug/ml LM and 4-HA with 200ug/ml LM. No significant difference were observed between groups

**Fig. 6:**
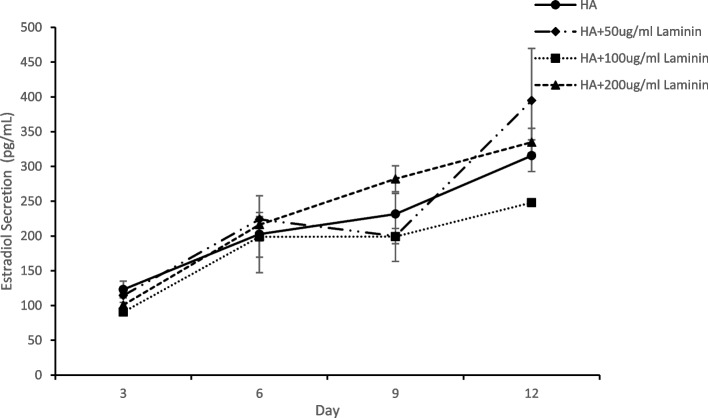
Comparison of estradiol secretion per follicle during 3-D culture in HA or HA with varying concentrations of laminin. Data is expressed as mean ± SD

Based on the above experiment, HA-LM appeared to be the most promising amongst the ECM products tested. In a follow up experiment, we contrasted follicle growth in HA gel with varying concentrations of laminin (Fig. 5). HA-LM at either 50 or 100 ug /ml worked well in conjunction with the HA gel in supporting follicle growth, oocyte ovulation and maturation. However, no advantage was observed with HA-LM over HA alone. Hoechst staining of GV oocytes before hCG trigger revealed very similar chromatin patterns between oocytes derived from HA alone or with laminin. The percentage of GV oocytes with the SN staining pattern was 94%, 73% and 78% for HA, HA-LM50 and HA-LM 100, respectively. Normal chromosome alignment and spindle configuration was observed in 64%, 55% and 58% of immunostained metaphase II oocytes from HA, HA-LM50 and HA-LM100, respectively. We also used polarized light to view meiotic spindles in living oocytes (unfixed). Retardance values measured for meiotic spindles in the groups did not differ (HA 2.03 ± 0.30, HA-LM50 2.05 ± 0.34, HA-LM100 1.96 ± 0.04), suggesting similar spindle birefringent characteristics. Estradiol secretion with different concentrations of laminin are shown in Fig. 6. Estradiol secretions in the HA-LM50 and LM-200 treatment were higher than HA alone but differences were not significant, and likely just reflected more proliferation of the granulosa cell compartment. It is not clear why HA-LM 100 was lower. Once again, no direct correlation was noted between estradiol levels in the treatment groups and oocyte maturation rate.

## Discussion

The focus of our current study was to expand on our original research describing the first application of a novel tyramine-linked hyaluronan gel for 3-D culture of follicles and oocyte maturation. This study documents the functional competence of oocytes derived from *in vitro* culture of preantral follicles in this HA biomatrix. The benchmarks selected to assess functional competence, namely meiotic maturation, ability to be fertilized and ability to blastulate form the basis of the mouse bioassay. These criteria are typically used to validate the use of any technique or culture medium with human oocytes or embryos.

The first outward sign of meiotic maturation in the developing oocyte is chromatin condensation into perinucleolar rings (SN pattern). This occurs at the late stage of oocyte growth and marks the end of the transcriptional phase [31]. The SN pattern appears to correlate with mouse GV oocyte growth, acquisition of meiotic competence and more importantly the ability to develop beyond the 2-cell stage to blastocyst [28, 32, 33]. Our HA culture model supported the growth and acquisition of nuclear as well as cytoplasmic competence by GV oocytes within preantral follicles. The chromatin organization in GV oocytes in HA-embedded follicles transformed during the *in vitro* culture period, going from the NSN to the SN configuration. Upon ovulation, a high percentage of these oocytes completed meiotic maturation to the metaphase II stage. These *in vitro* matured oocytes were capable not only of being fertilized but also of developing to the blastocyst stage, two well established indicators of functional competency.

Follicle encapsulation has been one of the most effective methods for maintaining spherical follicle architecture, preventing disruption of gap junctions and sequestering trophic factors around the growing oocyte/follicle complex(reviewed [16–18]. The most extensively studied biomaterial for use in follicle encapsulation and culture has been alginate, a polysaccharide isolated from brown algae [23, 26, 34, 35]. Mature oocyte ovulation however first requires the release of the encapsulated follicle from the bead by treatment with a chelating agent. Also, alginate unlike natural ECM lacks cell adhesion properties. To overcome these issues, researchers have tested alginate in combination with other materials such as Matrigel, fibrin and the cell adhesion peptide sequence RGD with the goal of enhancing cell binding, and promoting follicular expansion and oocyte maturation [25, 36, 37]. These studies suggest that alginate in combination with other biomaterials may be superior to alginate alone. 

The ultimate challenge and proof of concept for any IVM culture model is to demonstrate successful implantation, pregnancy, live births and health of offspring. Within the published literature on mouse IVM with different 3-D culture models, including the widely used alginate system remarkably few studies have transferred resultant embryos [34, 38, 39]. Amongst these studies live birth rates have been highly varied (0.5% to 20%), depending on maturity of follicles being cultured (i.e. primordial vs preantral), embryonic stage (pronuclear, 2-cell or blastocyst), site of transfer (oviduct vs uterus) as well as type of culture system. Xu and colleagues first reported live births with the alginate culture model [34]. In their study, a 68% fertilization rate was achieved with IVM oocytes from 16-day old pre-antral follicles. Normally fertilized pronuclear embryos were transferred to the oviducts of mice and resulted in 4 live offspring (4/20). Despite the higher fertilization rates in our present work (88%) working with 12-14 day old preantral follicles, the success rate in our pilot study was low (1/46). Due to its cannibalization, we cannot comment on the health of the delivered pup. *In vitro* growth of the pronuclear embryos to blastocyst rather than immediate transfer to the oviduct may partially explain our low birth rate. Early stage transfer has the advantage of allowing embryos derived from IVM oocytes to grow within an in vivo environment, with exposure to oviductal and uterine growth factors which may allow better synchrony with the endometrium, facilitating implantation. Certainly, with in vivo matured oocytes far higher birth rates (62-73%) have been reported with either uterine or transcervical embryo transfer [40]. One of the concerns with long term *in vitro* follicle growth is epigenetic regulation in oocytes and embryos by the culture environment and the long term effect on health of offspring [41–43]. Major changes and re-programming occur during gametogenesis and growth to a totipotent embryo. Perturbation of this process can potentially lead to abnormal fetal development.

Unlike alginate, hyaluronan (HA) is a naturally occurring glycosaminoglycan and the primary component of the extracellular matrix (ECM) found in animal as well as human tissue. It acts as a signaling molecule, promotes granulosa cell proliferation and induces expression of PGRMC1 which activates survival pathways preventing granulosa cell apoptosis [44]. Hyaluronan also plays a major role in organization of the ECM. This makes it a highly attractive choice for encapsulating ovarian follicles and even creating an “artificial ovary”. Laminin as well as ECM serve as scaffolding, sequester growth factors and affect cell differentiation. ECM’s extracted from basement membranes reflect the micro environment of the tissue cells they are derived from. Matrigel, a widely used scaffold for tissue culture originates from the basement membrane of a mouse sarcoma. Our rationale for testing placental ECM was that being of human reproductive origin and from a non-tumor source it might better mimic the native environment of somatic cells and be a better option for future human follicle work. Placental ECM in a 3-D culture model was shown to restore hair follicle regeneration by dermal papilla cells [45]. In our present work, neither type of ECM had a demonstrable positive effect on final meiotic maturation. HA alone proved to be entirely sufficient for supporting the development of mature competent metaphase II oocytes. This was a significant finding, reinforcing the value of this simple but effective HA bio matrix for 3-D culture of follicles. 

The creation of an “artificial ovary” or “organoid” using follicles is an exciting prospect. The translucent nature of the HA gel matrix and its moldability, facilitates the monitoring and culture of individual follicles, groups of isolated follicles as well as intact follicle clusters of varying sizes. This opens up the possibility of eventually using this type of HA gel for human ovarian tissue transplantation. An ECM based matrix for injectable tissue engineering that can be crosslinked in situ would be especially beneficial since it would facilitate use of laparoscopic techniques for in vivo placement of a matrix/follicle mix or else seeding of a scaffold for in vivo or *in vitro* use. In the present work, HRP/peroxide interaction was used for in situ cross linking of the tyramine-linked hyaluronan gel. This tyramine functionalized HA can also be crosslinked using visible light [46–48]. These cross-linking options allow many design possibilities for use of this matrix including bioprinting. Gel rigidity is easily tunable allowing sufficient elasticity for granulosa cell expansion. The ability to trigger ovulation without extracting the embedded follicles from the HA gel biomatrix may prove especially beneficial if follicles are to be used for in vivo tissue grafting.

Translation of *in vitro* culture work from the animal model to human ovarian tissue is difficult. Whereas the mouse model is good for initial testing of culture variables and matrices, the growth trajectory and timeline for mouse follicle development are much shorter. Human follicle growth from the primary to the antral follicle stage in vivo may take as long as 120 days, making it particularly challenging to design an *in vitro* culture model. For human follicle cultivation, multi-step protocols have proved necessary to grow primary follicles from the ovarian cortex [49]. Controlling rigidity of the biomatrix is believed to be essential for success with human follicles [50]. Despite over 30 years of research, progress has been slow and a live birth from human *in vitro* follicle culture and oocyte maturation remains elusive. Testing of new and different biomatrices with human follicles will aid in designing a more successful *in vitro* culture model.

In conclusion, the versatility, simplicity and ability to be used at various rigidities gives this new biomaterial numerous advantages. Our culture model yielded functionally competent oocytes after IVM, capable of developing to blastocyst. A limitation of this study was not having on-site facilities to allow extensive fresh blastocyst transfer experiments but having to instead rely on an outside lab. This study does however open up many new avenues of research. Cultivating follicle clusters in our 3-D system is an approach that needs further study with adult ovaries to determine if primary follicles can be recruited to grow. Spatial orientation, constraints imposed by the biomatrix, in addition to the *in vitro* microenvironment may need to be further optimized to provide all the biophysical and biochemical signaling required to nurture follicle growth. These requirements may differ between species. Whether this HA culture model can be applied to *in vitro* culture of human follicles or as a biomatrix for ovarian tissue transplantation remains to be seen.

## Data Availability

All data generated from animal experiments and analyzed during this study are included in this published article and available at the Cleveland Clinic Fertility Center Research Lab.
